# Quantitative Expression of *TYR, CD34*, and *CALD1* Discriminates Between Canine Oral Malignant Melanomas and Soft Tissue Sarcomas

**DOI:** 10.3389/fvets.2021.701457

**Published:** 2021-08-06

**Authors:** Mayra F. Tsoi, Tuddow Thaiwong, Rebecca C. Smedley, Erica Noland, Matti Kiupel

**Affiliations:** ^1^Veterinary Diagnostic Laboratory, College of Veterinary Medicine, Michigan State University, Lansing, MI, United States; ^2^Department of Pathobiology and Diagnostic Investigation, College of Veterinary Medicine, Michigan State University, East Lansing, MI, United States

**Keywords:** melanoma, soft tissue sarcoma, tyrosinase, CD34, caldesmon, *SOX10*, SOX-10, canine

## Abstract

Canine oral malignant melanomas (OMMs) exhibit a variety of morphologic phenotypes, including a spindloid variant. The microscopic diagnosis of spindloid OMMs is based on junctional activity and/or the presence of melanin pigment. In the absence of these features, spindloid OMMs are difficult to differentiate from soft tissue sarcomas (STS). An antibody cocktail (MDX) that includes Melan-A, PNL2, and tyrosinase-related proteins 1 and 2 (TRP-1 and TRP-2) is the current gold standard for identifying amelanotic OMMs by immunohistochemistry (IHC). However, MDX is less sensitive for diagnosing spindloid amelanotic OMMs. This raises concern for biopsy specimens that lack overlying epithelium, making it potentially difficult to differentiate OMM from STS by IHC. The goal of this study was to identify additional markers to help differentiate between STS and OMMs that lack pigment and junctional activity. SOX-10 has recently been proposed as a sensitive marker for melanocytes in humans but has not been validated in dogs. Similarly, RNA expression for various genes has been analyzed in humans, but not in the context of diagnosing canine melanocytic neoplasms. For this retrospective study, formalin-fixed, paraffin-embedded tissues from 20 OMMs, 20 STS, and 20 oral spindle cell tumors (OSCTs) that lacked junctional activity and pigmentation were selected. IHC for MDX, SOX-10, and laminin, in parallel with RT-qPCR of *TYR, SOX10, CALD1, CD34, DES*, and *LAMA1*, was performed in all cases. *TYR, CD34*, and *CALD1* were the most discriminatory genes in differentiating between OMM and STS, all having 100% specificity and 65, 95, and 60% sensitivity, respectively. While all 20 OMMs were immunohistochemically labeled for SOX-10, two STS were also labeled (100% sensitivity and 90% specificity). MDX IHC labeled all 20 OMMs and no STS. Surprisingly, none of the 20 OSCTs expressed *TYR* RNA above the cutoff, and 14/20 OSCTs expressed *CALD1* or *CD34* RNA above the cutoff, thereby confirming them as STS. Four OSCT were suspect STS, and no OSCTs were confirmed as OMMs based on IHC and RNA expression patterns. In conclusion, the RNA levels of TYR, CD34, and CALD1 should be evaluated in suspected amelanotic OMMs that are negative for MDX to accurately differentiate between OMM and STS.

## Introduction

Oral malignant melanomas (OMMs) are the most common malignant oral neoplasm in dogs (1–3). They are locally invasive and have a high rate of metastasis to local lymph nodes and to the lungs (1, 2). An overall median survival time of 6 months has been reported (from surgical removal to time of death) (1, 2, 4). The mean age at diagnosis is 10–11 years of age, and certain breeds appear to be overrepresented, including poodles, golden and Labrador retrievers, Rottweilers, Yorkshire terrier, cocker spaniels, chow-chows, Scottish terriers, and dachshunds (2). Canine OMMs exhibit variable morphologic phenotypes, including epithelioid, spindloid, mixed, whorled, balloon cell, signet ring cell, clear cell, and adenoid cell types (5). In dogs, OMMs most commonly present, similarly to human mucosal melanomas, with an intraepithelial component (junctional activity), lentiginous spread, and extensive subepithelial vertical growth that causes the actual mass effect (5, 6). The spindloid phenotype represents 34% of OMMs according to Ramos-Vara et al. (7) and is diagnosed histologically based on the presence of melanin pigment and junctional activity. In the absence of these morphologic features, spindloid amelanotic OMMs are difficult to differentiate from oral soft tissue sarcomas (STS) microscopically (8). Such distinction is important as STS, in contrast to OMMs, rarely metastasize and are primarily treated with surgery and/or radiation therapy with a significantly longer reported median survival time of 540 days (8). While some antibodies such as Melan-A and PNL2 have been shown to be highly specific in differentiating OMMs from STS, other antibodies with high sensitivity for detecting OMMs, such as MITF-1 and S100, have poor specificity and also label a large percentage of STS (3). Currently, the most sensitive and specific method to diagnose amelanotic OMMs in dogs is immunohistochemical labeling with a cocktail of four antibodies: Melan-A, PNL2, and tyrosinase-related proteins 1 and 2 (TRP-1 and TRP-2) (3). We previously demonstrated that this antibody cocktail has 100% specificity and 93.9% sensitivity in detecting canine oral melanocytic neoplasms compared to STS (3). In that study, the spindloid phenotype was the least likely variant to label with the individual specific melanocytic markers and the antibody cocktail. In three out of nine cases of spindloid OMMs, the antibody cocktail did not label the spindle cell component in the subepithelial portion of the mass (vertical growth portion). Furthermore, intraepithelial neoplastic cells are the most likely neoplastic cells in OMMs to label with melanocytic markers as they are the most differentiated cells (3). As the epithelium overlying canine oral malignancies is commonly ulcerated, surgical biopsies of spindloid amelanotic OMMs often present a challenge in routine diagnostics. The true incidence of OMMs within this specific group of oral spindle cell tumors (OSCTs) that lack pigmentation and junctional activity is unknown, and identification of novel markers capable of differentiating between OMMs and STS would advance our diagnostic capabilities.

Antigens such as CD34, SOX-10, caldesmon, laminin, and desmin have been shown to be expressed in various types of STS in dogs (9); however, their utility to differentiate spindloid amelanotic OMMs from oral STS in dogs has not been studied in detail. In human melanomas, not only has the expression of melanoma-associated proteins been investigated with immunohistochemistry (IHC) but also numerous studies have investigated gene expression patterns for their diagnostic and prognostic utility (10, 11). The expression of tyrosinase has been especially investigated as it is both immunogenic and essential in key processes of melanogenesis (12). The expression of *TYR* mRNA has been detected in 100% of investigated human melanoma cell lines in some studies (10), and others found that high mRNA levels of *TYR* in human metastatic melanomas were predictive of overall improved survival (11). Similar studies have not been conducted in dogs. The goal of this study was to investigate the RNA and protein expression of melanoma-associated markers, as well as markers associated with STS, to establish expression patterns for these two entities and, ultimately, to accurately differentiate spindloid amelanotic OMMs from non-melanocytic OSCTs.

## Materials and Methods

### Case Selection

**Figure 1:**
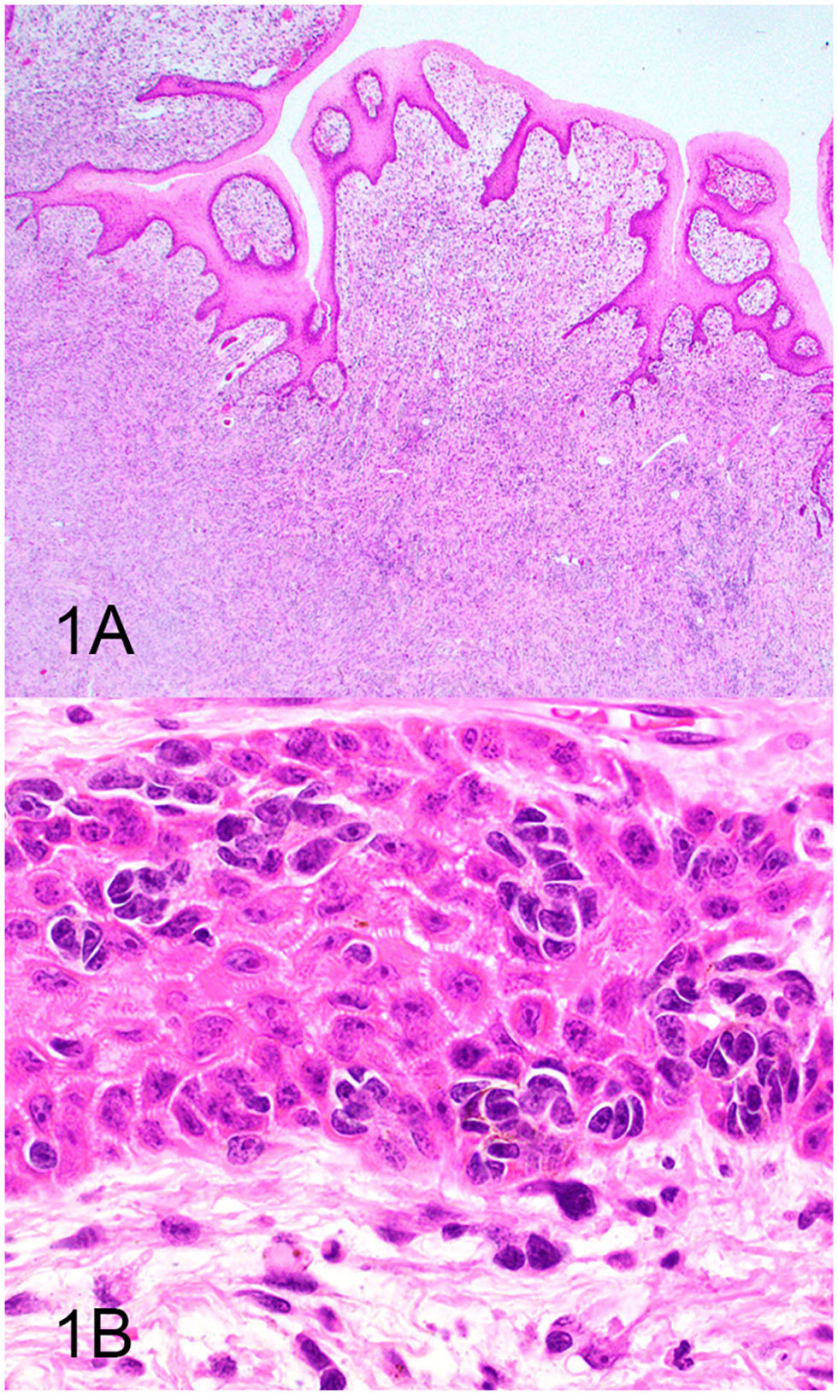
Oral malignant melanoma (OMM), hematoxylin and eosin staining. **(A)** OMM with junctional activity and composed predominantly of subepithelial spindloid cells with <5% containing pigment. Magnification, ×4. **(B)** Higher magnification of **(A)** highlighting junctional activity (intraepithelial nests within the basal layer of the epithelium). Magnification, ×40.

**Figure 2:**
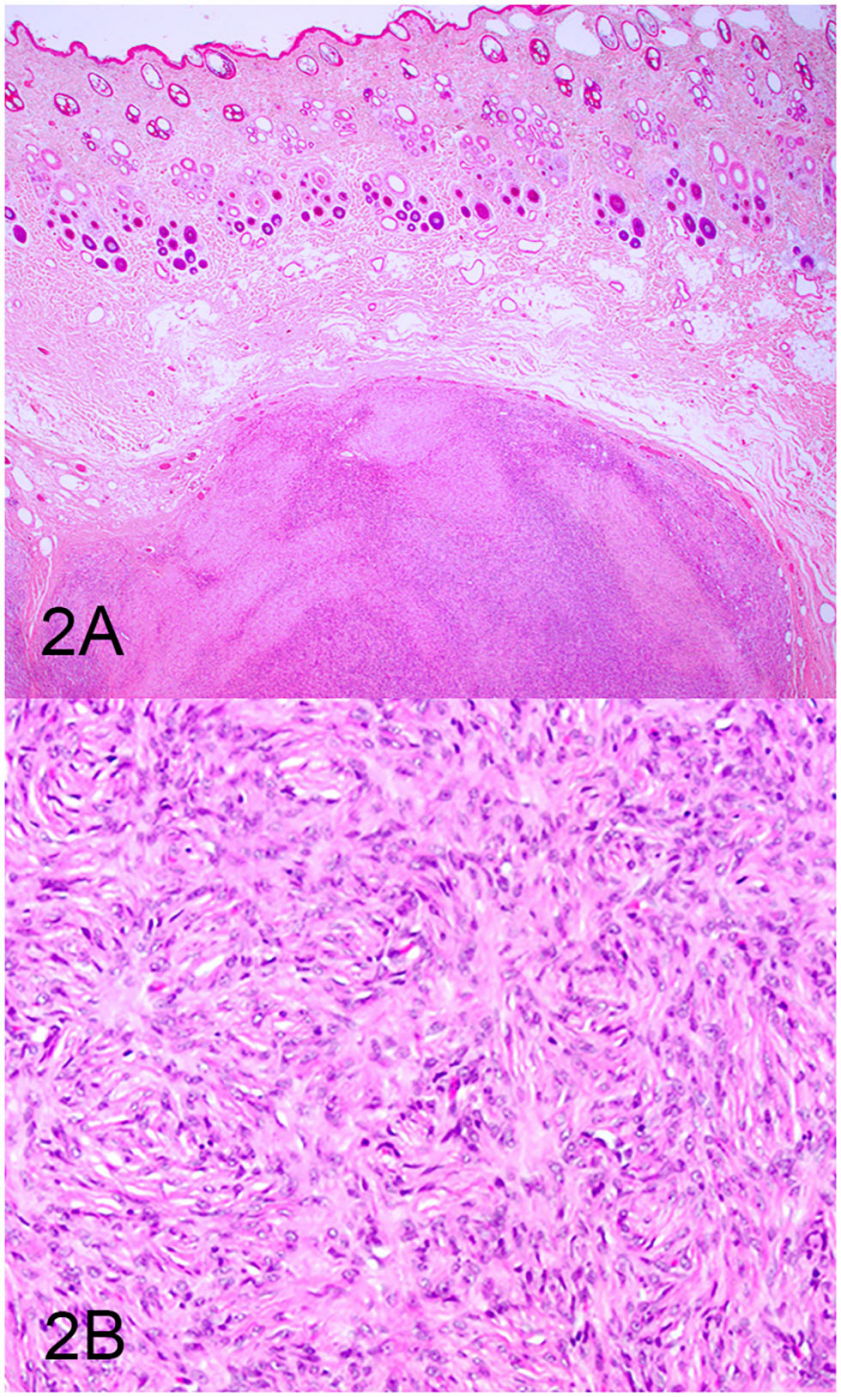
Soft tissue sarcoma (STS), hematoxylin and eosin staining. **(A)** STS composed of sheets of spindloid cells. Magnification, ×4. **(B)** Higher magnification of **(A)** highlighting spindle cells arranged in a fingerprint pattern. Magnification, ×20.

**Figure 3:**
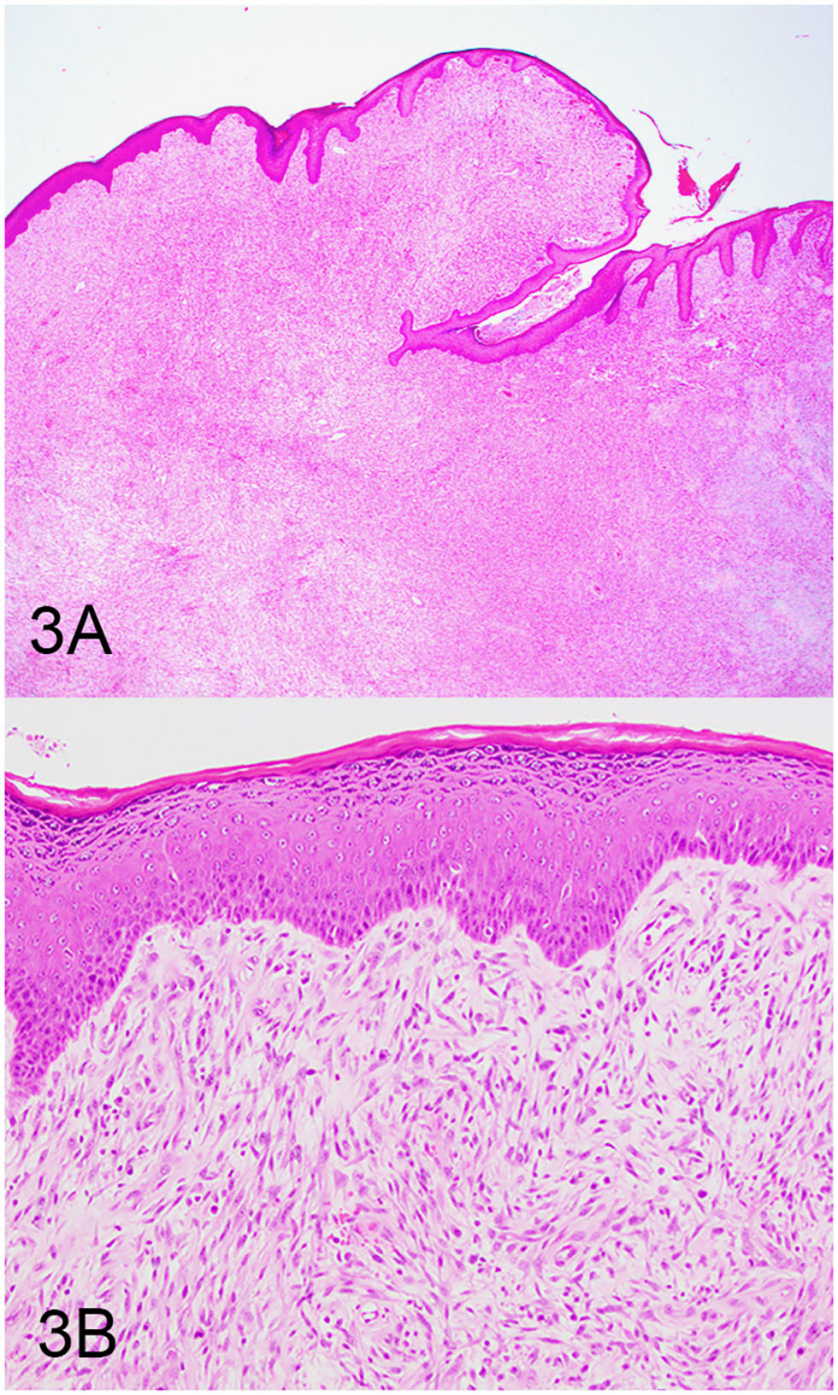
Oral spindle cell tumor (OSCT), hematoxylin and eosin staining. **(A)** OSCT with no junctional activity, but the neoplastic cells are present at the epithelial–subepithelial junction, are predominantly spindloid, and have no pigment. Magnification, ×4. **(B)** Higher magnification of **(A)** highlighting neoplastic cells at the epithelial–subepithelial junction. Magnification, ×20.

Three groups, including OMMs, subcutaneous STS, and OSCTs, of 20 cases each were selected from the Michigan State University Veterinary Diagnostic Laboratory archives of formalin-fixed, paraffin-embedded tissues that had been submitted for routine surgical biopsy between 2008 and 2020. All samples had been fixed in 10% neutral buffered formalin, routinely processed, embedded in paraffin wax, sectioned at 5 μm, and routinely stained with hematoxylin and eosin. Diagnoses were independently established for all cases through review by two board-certified pathologists (MK and TT). The first group (OMM group) included 20 dogs with OMMs (Figures 1A,B) that were predominantly spindloid and exhibited junctional activity but were poorly pigmented (<5% of neoplastic cells). All cases in this group were positive for the melanoma diagnostic antibody cocktail (MDX) that contains antibodies against Melan-A, PNL2, TRP-1, and TRP-2. The second group (STS group) included 20 dogs with subcutaneous STS that were immunohistochemically negative for MDX and exhibited features of malignant fibrous tumors, malignant nerve sheath tumors, and malignant perivascular wall origin tumors (Figures 2A,B). The last group (OSCT group) included 20 dogs with OSCTs (Figures 3A,B), defined as being predominantly composed of spindloid neoplastic cells that had no pigmentation, that were negative for MDX and that were present at the epithelial–subepithelial junction but lacked junctional activity (neoplastic cells were not identified within the basal layer of the epithelium). These neoplasms were all classified as malignant based on their high cellularity, poor degree of differentiation, and invasion into the adjacent stroma.

### RNA Extraction and RT-qPCR

Regions of high tumor cellularity (at least 80%), away from the overlying epithelium, from inflammation, and from necrosis, were selected (manually shaved) for RNA extraction from each case. Total RNA was isolated from formalin-fixed, paraffin-embedded tissue using RecoverAll™ Total Nucleic Acid Isolation Kit (ThermoFisher Scientific, catalog#AM1975) according to the protocol of the manufacturer. Following deparaffinization with CitroSolv, tumor tissue was incubated in proteinase K containing lysis buffer. RNA was quantified using Qubit (ThermoFisher Scientific, catalog#Q32852). Six hundred nanograms of total RNA was treated with TURBO DNA-free™ (ThermoFisher Scientific, catalog#AM1907) to remove contaminating DNA. First-strand cDNA synthesis was performed using SuperScript III Reverse Transcriptase (ThermoFisher Scientific, catalog#18080044) with random primers (Promega, catalog#C1181). The cDNA was then column-purified by using QIAquick PCR Purification Kit (QIAGEN, catalog# 28104) and eluted with distilled nuclease-free water at 5 ng/μl. For the determination of specific gene expression, each primer was designed with Primer3 software (13, 14). The primers for *TYR, SOX10, CALD1, CD34, DES*, and *LAMA1* are listed in Supplementary Table 1. Ten nanograms of cDNA was used as the template in the reaction mixture for quantitative real-time PCR, using SYBR Green (ThermoFisher Scientific, catalog# 4309155) according to the recommendations of the manufacturer. The protocol was as follows: initial denaturation at 95°C for 30 s, followed by 40 cycles of denaturation at 95°C for 5 s, annealing at a temperature suitable for each gene marker for 10 or 20 s, and extension at 72°C for 10 s. The baseline was set automatically, and the threshold Ct was defined as the number of cycles in which the fluorescence exceeded the automatically set threshold. A normal gingival tissue sample was chosen as a calibrator, and the ratio of each target gene to the beta-2 microglobulin expression for each tumor sample was normalized by the same ratio for the normal tissue sample using the delta-delta Ct (ΔΔCt) method. Each sample was assayed in triplicate. A control and a reference were included in every run.

### Immunohistochemistry

Routine immunohistochemical labeling was performed as previously described (3) using MDX (which contains antibodies against Melan-A, PNL2, TRP-1, and TRP-2) and antibodies against SOX-10, laminin, desmin, and S100 (Supplementary Table 2). IHC for MDX, SOX-10, and laminin was performed on all three groups. In addition, IHC for desmin was performed on STS and OSCTs only, and IHC for S100 was performed on OSCTs only. For desmin, laminin, and S100, IHC was performed on a Bond autostainer automated system using BOND Polymer Refine Detection kit (Leica Microsystems). For MDX and SOX-10, IHC was performed on Dako Omnis using EnV FLEX HRP Magenta (Agilent). Positive and negative controls for each antibody were used appropriately in every run (Supplementary Table 2). IHC for tyrosinase was attempted using two different antibodies but was unsuccessful (data not shown).

### Histological Examination

Immunohistochemically labeled sections were scored as previously described (3) by two board-certified pathologists (RS and MK). Briefly, percentages of positively labeled neoplastic cells were semi-quantitatively scored using the following cutoffs: ≤10% (considered negative and identified as *N*), 11–50% (assigned a score of 1), 51–80% (assigned a score of 2), or 81–100% (assigned a score of 3). For MDX, neoplasms were also considered positive if intraepithelial nests, consisting of at least five clustered neoplastic cells, labeled positively or if there were aggregates of at least 20 neoplastic cells that labeled positively anywhere within the neoplasm as previously described (3). The sensitivity of SOX-10 IHC as a melanocytic marker was determined based on the ability of the antibody to positively label the 20 OMMs, and specificity was determined based on the absence of labeling of the 20 STS, which served as negative controls.

### Statistical Analyses

Data are presented as means ± SEM. The differential expression of OMM relative to STS genes was analyzed by Student's *t*-test. Cutoff values for relative RNA levels were determined using receiver operating characteristic (ROC) curve. Sensitivity was determined based on the number of cases (OMM or STS) above the established cutoff value; specificity was determined based on the number of (OMM or STS) cases below the established cutoff value. A value of *P* ≤ 0.05 was considered statistically significant. Analyses were performed using Prism software, v.6.01 (GraphPad Software).

## Results

**Figure 4:**
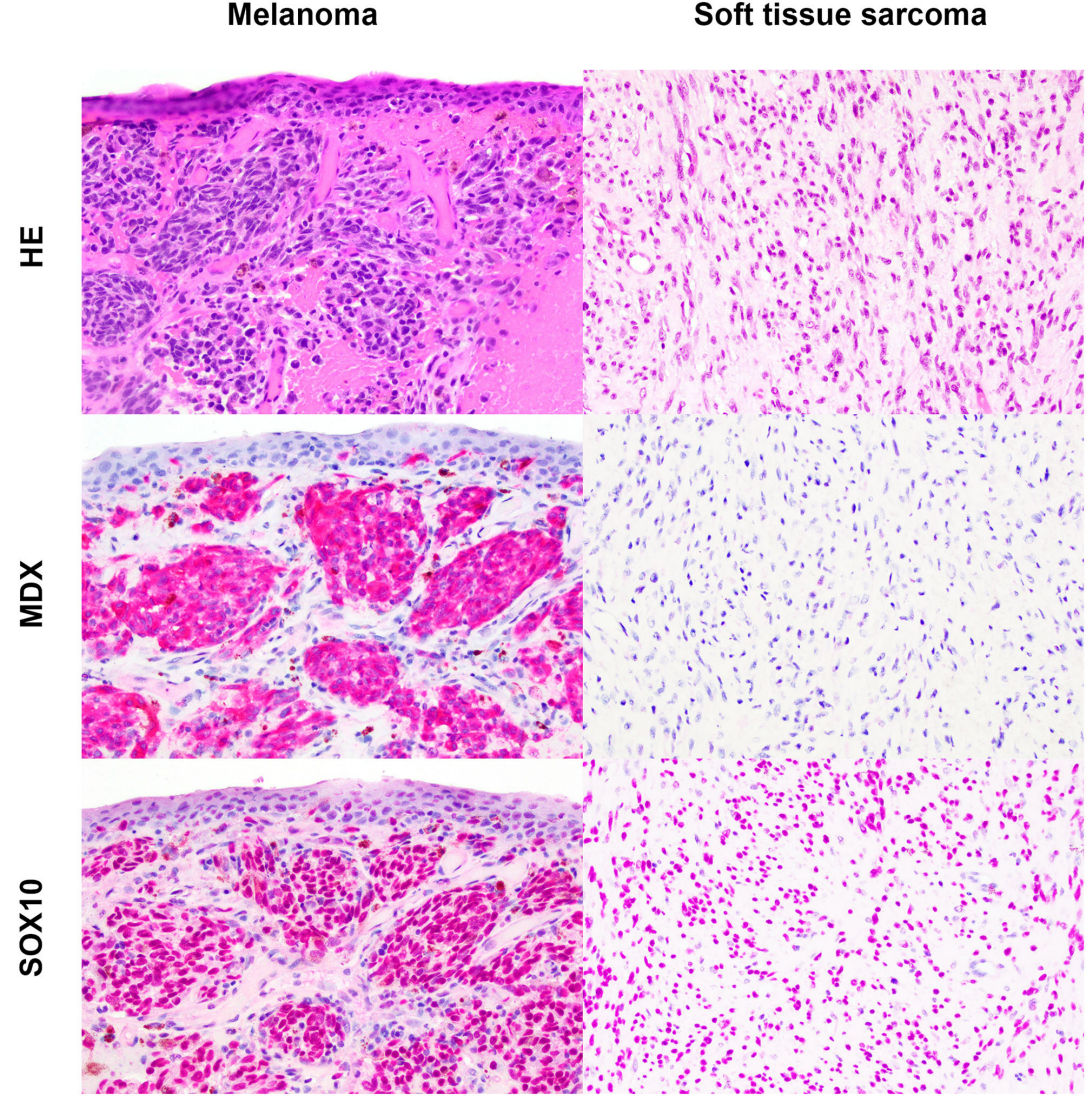
Oral malignant melanoma (OMM) and soft tissue sarcoma (STS) stained with hematoxylin and eosin or immunohistochemically labeled with the melanoma immunodiagnostic cocktail (MDX; red cytoplasmic labeling) or SOX-10 (red nuclear labeling) with hematoxylin counterstaining. OMM with red cytoplasmic labeling for MDX with a score of 3 and red nuclear labeling for SOX-10 with a score of 3. STS negative for MDX and red nuclear labeling for SOX-10 with a score of 3. Magnification, ×20.

**Figure 5:**
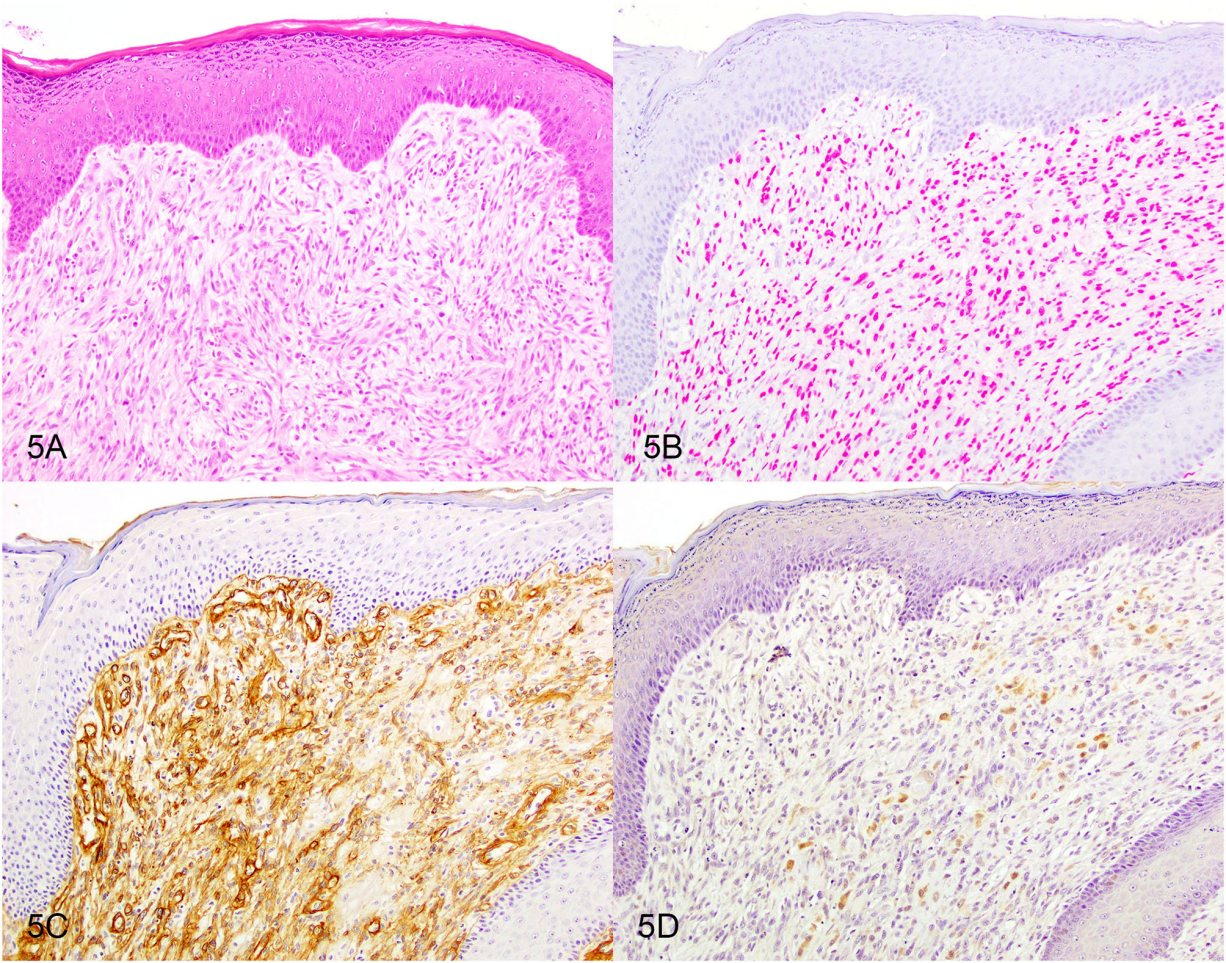
Oral spindle cell tumor. Serial sections stained with **(A)** hematoxylin and eosin or immunohistochemically labeled for **(B)** SOX-10 showing red nuclear labeling or for **(C)** laminin showing brown basement membrane labeling or for **(D)** S100 showing brown cytoplasmic labeling. Magnification, ×20.

Based on the selection criteria, MDX IHC correctly labeled all 20 OMMs and did not label any of the 20 STS and none of the 20 OSCT (Figure 4; Table 1). In two OMM cases, MDX IHC labeling was limited to the intraepithelial nests of neoplastic melanocytes and was absent in the subepithelial neoplastic spindloid melanocytes. SOX-10 IHC labeling was also seen in all 20 OMMs, but two STS labeled as well (100% sensitivity and 90% specificity in diagnosing OMM). Six of the OSCTs labeled for SOX-10 (Figure 5; Table 2). IHC labeling for laminin was detected in the basement membranes surrounding neoplastic cells in six OMMs and 18 STS (90% sensitivity and 70% specificity in diagnosing STS). Only one STS had cytoplasmic labeling for desmin. Sixteen OSCTs labeled for laminin and two for desmin (Figure 5). In addition, five OSCTs labeled for S100.

**Table 1:** Number of cases with immunohistochemical expression of tested antibodies in oral malignant melanomas (OMM) and soft tissue sarcomas (STS).

	**OMM**			**STS**			
	**MDX**	**SOX-10**	**Laminin**	**MDX**	**SOX-10**	**Laminin**	**Desmin**
Epi only^a^	2	0	0	NA	NA	NA	NA
≤10%^b^	0	0	14	20	18	2	19
11–50%	5	0	4	0	0	10	1
51–80%	7	3	2	0	1	6	0
81–100%	6	17	0	0	1	2	0
Total # positive	20	20	6	0	2	18	1

*NA, not applicable*.

a *Expression limited to intraepithelial nests*.

b *Neoplasms with ≤10% of cell labeling were considered negative*.

**Table 2:** Number of cases with immunohistochemical expression of tested antibodies in oral spindle cell tumors (OSCT).

	**MDX**	**SOX-10**	**Laminin**	**Desmin**	**S100**
≤10%^a^	20	14	4	18	15
11–50%	0	1	10	0	2
51–80%	0	0	3	0	2
81–100%	0	5	3	2	1
Total number of positive	0	6	16	2	5

a *Neoplasms with ≤10% of cell labeling were considered negative*.

**Figure 6:**
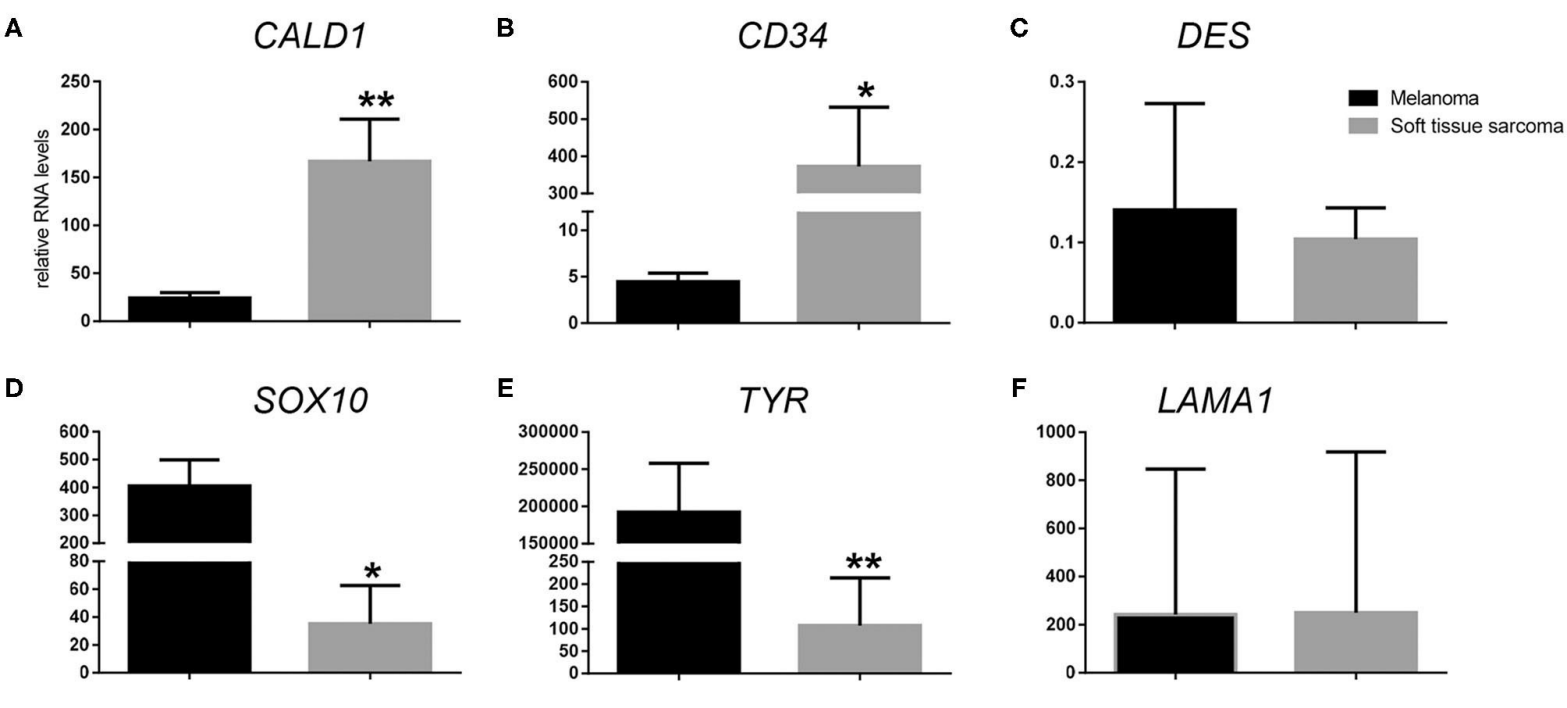
Relative RNA expression levels of **(A)** *CALD1*, **(B)** *CD34*, **(C)** *DES*, **(D)** *SOX10*, **(E)** *TYR*, and **(F)** *LAMA1* in oral malignant melanoma vs. soft tissue sarcoma. *p < 0.05, **p < 0.01.

Following RT-qPCR for *TYR, SOX10, LAMA1, DES, CD34*, and *CALD1*, the cutoff values for relative RNA levels were determined using ROC curves (Table 3). Thirteen OMMs expressed *TYR* RNA levels above the cutoff value, all 20 OMMs expressed *SOX10* RNA above the cutoff value, and six and one OMM expressed *LAMA1* RNA and *DES* RNA above the cutoffs, respectively (Table 4). None of the OMMs expressed *CD34* RNA or *CALD1* RNA above the cutoffs. In contrast, none of the STS expressed *TYR* RNA levels above the cutoff value, and only three STS expressed *SOX10* RNA above the cutoff (Table 5). The two STS cases with the highest *SOX10* RNA expression also labeled for SOX-10 with IHC. All except one STS expressed *CD34* RNA above the cutoff. *CALD1* and *DES* RNA levels above the cutoffs were detected in 12 and seven STS, respectively. There was a statistically significant difference in the RNA expression levels of select genes in OMM relative to STS (Figure 6) for *TYR* (192,538 ± 65,305 vs. 107.5 ± 106.7; *p* < 0.05), *SOX10* (405.5 ± 93.65 vs. 35.14 ± 27.53; *p* < 0.05), *CD34* (4.462 ± 0.9124 vs. 372.9 ± 159.2; *p* < 0.05), and *CALD1* (24.21 ± 5.784 vs. 166.7 ± 43.97; *p* < 0.01). No significant difference in RNA expression levels was identified between OMMs and STS for *LAMA1* (241.8 ± 135.1 vs. 249.3 ± 149.4) and *DES* (0.1405 ± 0.1327 vs. 0.1040 ± 0.03911). Based on these data, the specificity and the sensitivity of *TYR* RNA levels for detecting OMM were 100 and 65%, respectively (Table 3). The specificity of *CD34* and *CALD1* RNA levels above the cutoff level for detecting STS was also 100%, and the sensitivity for these tests was 95 and 60%, respectively. In contrast, *SOX10* RNA levels had a specificity of only 85% for detecting OMM but a sensitivity of 100%. When analyzing the OSCT group by RT-qPCR, none of the cases expressed *TYR* RNA above the cutoff level, but 14 OSCTs expressed either *CD34* or *CALD1* RNA, consistent with a diagnosis of STS (Table 6). Of the remaining six OSCTs, three (#2, #18, and #19) expressed *SOX10* RNA above the cutoff level. Of these three OSCTs, one (#19) expressed *DES* RNA and was positive for laminin and negative for S100, desmin, and MDX by IHC. *SOX10* RNA was also expressed above the cutoff level in two OSCTs that had either *CD34* or *CALD1* RNA levels above the cutoffs (#1 and #20). In total, five OSCT cases expressed *DES* RNA and seven OSCT expressed *LAMA1* RNA above the cutoff. A final diagnosis for each OSCT was made based on the combined RNA expression and immunohistochemical labeling results. Fourteen OSCTs were diagnosed as STS based on RNA levels above the cutoffs for either *CD34* or *CALD1*, no expression of *TYR* RNA, and lack of labeling for MDX by IHC. Two of these 14 OSCTs labeled positively by IHC for desmin and five for S100, suggesting a differentiation toward pericytes or smooth muscle cells and nerve sheath cells, respectively. Two of the remaining six OSCTs were diagnosed as undifferentiated malignant neoplasms, as they lacked expression and labeling patterns that supported either an OMM or STS. The remaining four OSCTs were diagnosed as suspect STS. While three of those OSCTs had RNA expression levels of *SOX10* above the cutoff and immunohistochemically labeled for SOX-10, one OSCT also had RNA expression levels for *DES* above the cutoff, and all four OSCTs were immunohistochemically positive for laminin and negative for MDX and did not express *TYR* RNA, making a diagnosis of STS more likely.

**Table 3:** Cutoff values for RNA expression levels of each gene determined by receiver operating characteristic curve to establish sensitivity and specificity.

	**Cutoff (arbitrary units)**	**Sensitivity (%)**	**Specificity (%)**
SOX10^a^	19.4	100	85
TYR^a^	9,709	65	100
CD34^b^	15.9	95	100
CALD1^b^	90.0	60	100
DES^b^	0.065	35	95
LAMA1^b^	66.2	30	70

a *Sensitivity and specificity were calculated for oral malignant melanoma (OMM) relative to soft tissue sarcoma (STS)*.

b *Sensitivity and specificity were calculated for STS relative to OMM*.

**Table 4:** Relative RNA levels and immunoreactivity of oral malignant melanoma.

	**RT-qPCR (relative RNA levels)**						**IHC (score ***N***, 1, 2, 3)^a^**		
	**CALD1**	**CD34**	**DES**	**LAMA1**	**SOX10**	**TYR**	**MDX**	**SOX-10**	**Laminin**
1	50	2.7	ND	15	585	1,014,163	3	3	*N*
2	42	11.2	0.06	239	1,788	29	1	3	2
3	6	0.8	ND	62	268	17,284	1	3	1
4	7	2.1	0.02	264	196	ND	3	3	*N*
5	12	2.9	ND	ND	421	372,535	2	3	*N*
6	24	13.0	ND	ND	279	467,252	2	3	*N*
7	23	11.4	ND	240	239	884,650	3	2	*N*
8	4	1.2	0.01	20	133	149,618	2	3	*N*
9	4	2.2	ND	66	314	235,907	2	3	*N*
10	14	3.2	ND	ND	29	ND	Epi	2	1
11	12	9.6	0.05	477	376	238,549	3	3	*N*
12	5	2.7	ND	8	154	ND	2	3	*N*
13	14	5.9	ND	2,691	545	103,153	2	3	1
14	41	9.7	ND	ND	176	49,260	3	3	*N*
15	22	1.5	2.66	664	964	191,577	3	3	*N*
16	89	1.8	ND	ND	318	ND	Epi	3	*N*
17	9	2.4	ND	ND	62	ND	1	3	*N*
18	89	1.8	ND	3	131	44,317	1	2	1
19	14	ND	ND	24	988	82,463	2	3	*N*
20^b^							1	3	2
20^b^	3	3.2	0.01	61	142	ND			

*Gray background, RNA expression above cutoff or positive immunohistochemical labeling; ND, not detected; Epi, positive labeling was exclusively within the mucosal epithelium and thus precluded scoring*.

a *Percentages of positive cells were assigned a score of N (≤10% positive labeling of neoplastic cells), 1 (11–50%), 2 (51–80%), or 3 (81–100%)*.

b *Tissue in paraffin block was depleted, and another block from the same tumor was used for RT-qPCR*.

**Table 5:** Relative RNA levels and immunoreactivity of soft tissue sarcoma.

	**RT-qPCR (relative RNA levels)**						**Immunohistochemistry (score ***N***, 1, 2, 3)^a^**			
	**CALD1**	**CD34**	**DES**	**LAMA1**	**SOX10**	**TYR**	**MDX**	**SOX-10**	**Laminin**	**Desmin**
1	91	18.7	0.01	8	ND	2,134	*N*	*N*	1	*N*
2	547	903.8	0.06	66	ND	ND	*N*	*N*	1	*N*
3	54	127.5	0.02	ND	1	ND	*N*	*N*	1	*N*
4	33	95.1	ND	64	ND	ND	*N*	*N*	1	*N*
5	727	895.5	0.07	ND	41	ND	*N*	*N*	1	*N*
6	118	254.8	0.03	ND	ND	ND	*N*	*N*	1	*N*
7	16	120.5	0.01	26	1	ND	*N*	*N*	1	*N*
8	279	137.6	ND	1,416	1	ND	*N*	*N*	2	*N*
9	415	3,192.8	0.36	36	10	ND	*N*	*N*	1	*N*
10	97	29.3	ND	ND	548	ND	*N*	3	3	*N*
11	51	117.6	0.48	121	ND	ND	*N*	*N*	1	*N*
12	15	5.8	ND	115	100	ND	*N*	2	3	*N*
13	153	85.9	0.08	2,762	ND	ND	*N*	*N*	*N*	*N*
14	124	68.7	ND	202	ND	16	*N*	*N*	*N*	*N*
15	40	318.2	0.11	23	ND	ND	*N*	*N*	1	*N*
16	342	304.7	0.02	125	ND	ND	*N*	*N*	2	*N*
17	106	396.0	0.19	3	ND	ND	*N*	*N*	2	*N*
18	40	303.4	0.61	7	ND	ND	*N*	*N*	2	1
19	28	25.0	0.02	8	ND	ND	*N*	*N*	2	*N*
20	58	57.2	0.01	2	ND	ND	*N*	*N*	2	*N*

*Gray background, RNA expression above cutoff or positive immunohistochemical labeling; ND, not detected*.

a *Percentages of positive cells were assigned a score of N (≤10% positive labeling of neoplastic cells), 1 (11–50%), 2 (51–80%), or 3 (81–100%)*.

**Table 6:** Relative RNA levels and immunoreactivity of oral spindle cell tumors.

	**RT-qPCR (relative RNA levels)**						**Immunohistochemistry (score ***N***, 1, 2, 3)^a^**					**Diagnosis^b^**
	**CALD1**	**CD34**	**DES**	**LAMA1**	**SOX10**	**TYR**	**MDX**	**SOX-10**	**Laminin**	**Desmin**	**S100**	
1	219	7.5	0.01	38	236	ND	*N*	3	3	*N*	*N*	STS
2	53	4.1	0.03	44	704	ND	*N*	3	2	*N*	*N*	Suspect STS
3	125	98.7	ND	42	ND	231	*N*	*N*	2	*N*	2	STS
4	4	2.1	ND	77	ND	ND	*N*	*N*	*N*	*N*	*N*	UMN
5	219	3.7	0.46	6	ND	229	*N*	*N*	1	*N*	*N*	STS
6	57	16.4	0.73	571	ND	ND	*N*	*N*	1	3	*N*	STS
7	397	24.3	4.43	82	ND	7	*N*	*N*	1	3	*N*	STS
8	79	22.3	ND	ND	ND	ND	*N*	*N*	1	*N*	*N*	STS
9	168	24.6	0.01	16	2	9	*N*	*N*	1	*N*	*N*	STS
10	70	58.2	0.01	134	ND	ND	*N*	*N*	1	*N*	3	STS
11	9	37.3	ND	ND	ND	ND	*N*	*N*	*N*	*N*	*N*	STS
12	35	14.5	0.01	58	ND	ND	*N*	*N*	1	*N*	*N*	UMN
13	21	19.2	0.05	6	ND	82	*N*	*N*	*N*	*N*	*N*	STS
14	10	3.5	0.04	19	10	1	*N*	1	1	*N*	*N*	Suspect STS
15	34	21.0	0.16	71	ND	ND	*N*	*N*	1	*N*	*N*	STS
16	111	112.5	ND	530	ND	ND	*N*	*N*	2	*N*	1	STS
17	12	84.6	ND	ND	ND	ND	*N*	*N*	*N*	*N*	1	STS
18	7	4.0	ND	47	195	18	*N*	3	3	*N*	*N*	Suspect STS
19	12	10.1	0.11	ND	56	ND	*N*	3	1	*N*	*N*	Suspect STS
20	11	16.0	ND	114	79	ND	*N*	3	3	*N*	2	STS

*Gray background indicates RNA expression above cutoff or positive immunohistochemical labeling. ND, not detected; STS, soft tissue sarcoma; UMN, undifferentiated malignant neoplasm with no gene expression or immunohistochemical features of STS or oral malignant melanoma*.

a *Percentages of positive cells were assigned a score of N (≤10% positive labeling of neoplastic cells), 1 (11–50%), 2 (51–80%), or 3 (81–100%)*.

b *Final diagnosis based on RNA expression and immunohistochemical labeling*.

## Discussion

Canine oral spindloid amelanotic malignant neoplasms present a diagnostic challenge for veterinary pathologists. As junctional activity and pigmentation are two of the most distinct diagnostic features of OMMs, tissues submitted for surgical biopsy that lack surface epithelium and pigmentation often require additional molecular testing for an accurate diagnosis. In this study, we were able to demonstrate that analysis of the expression of *TYR, CALD1*, and *CD34* RNA accurately differentiated STSs from OMMs. *TYR* encodes one of the most studied melanin enzymes that plays a crucial role in the early steps of melanin synthesis and has been detected in 100% of investigated human melanomas in some studies (8). The proteins encoded by *CD34* and *CALD1* have been shown to be expressed in various types of STS in dogs, but not melanocytic neoplasms (3, 7, 13). Furthermore, based on the results of this study, the immunodiagnostic MDX cocktail containing antibodies against Melan-A, PNL2, TRP-1, and TRP-2 remains the gold standard for accurately differentiating OMMs from STS and other undifferentiated malignant neoplasms in a routine diagnostic setting. While two of the cases in the OMM group could not be confirmed as OMM by IHC without inclusion of the overlying epithelium, the MDX cocktail was 100% specific and did not label any STS or OSCT in this study as defined by the selection criteria. We had hypothesized that some of the OSCTs could represent true OMMs that simply did not label with MDX IHC in the sections that were available for examination. Unexpectedly, this appears unlikely based on our results, especially the RNA expression analysis results, that any of the 20 OSCTs represent an OMM. A total of 14 of 20 OSCTs were instead confirmed as STS based on their RNA expression pattern, as these tumors had levels of *CALD1* or *CD34* RNA above the cutoff values and lacked the expression of *TYR* RNA. Of the remaining six OSCTs, all were negative for *TYR* RNA and lacked immunohistochemical labeling for S100, which has been reported to be commonly expressed in undifferentiated OMMs (7). One of these six OSCTs labeled strongly for laminin by IHC (in ≥81% of neoplastic cells), and a similar strong IHC labeling for laminin was not observed in OMMs in this study. Another one of these OSCTs expressed *DES* RNA levels above the cutoff, which is more consistent with a diagnosis of STS rather than OMM. Lastly, three of these six cases had *SOX10* RNA levels below the cutoff, making a diagnosis of OMM highly unlikely. Thereby, it is highly likely that 18/20 OSCTs in this study represented STS. Only two of the 20 OSCTs could not be classified as OMM or STS based on their RNA expression patterns and immunophenotyping and were diagnosed as undifferentiated malignant neoplasms. However, it is highly unlikely that these neoplasms represent undifferentiated OMMs based on the absence of expression of both *TYR* and *SOX10* RNA as well as the lack of immunolabeling for MDX and SOX-10. These data clearly indicate that the incidence of STS among canine oral spindloid amelanotic neoplasms that lack junctional activity is significantly higher than assumed and that such cases require additional testing to avoid misdiagnosis as an OMM.

Intraepithelial nests of neoplastic melanocytes tend to represent the most differentiated stage of neoplastic melanocytes, and the inclusion of intact overlying epithelium in biopsies is essential for making an accurate diagnosis of spindloid amelanotic OMM (3, 15). While the MDX IHC cocktail has been shown to be highly sensitive and specific in detecting OMMs, we propose, based on the data presented here, that for OSCT cases that lack surface epithelium and that are MDX-negative, the relative expression levels of *TYR, CD34*, and *CALD1* RNA should be evaluated to discriminate between OMMs and STS.

SOX-10 is a transcription factor essential for neural crest and peripheral nervous system development and the formation of melanocytes (16). It has recently been used as a diagnostic IHC marker for human melanocytic neoplasms, as it is highly sensitive for detecting such tumors, including spindloid and desmoplastic subtypes (17). However, SOX-10 is essentially a pan-Schwannian and melanocytic marker (18). It commonly labels human peripheral nerve sheath tumors, including neurofibromas (95–98%), schwannomas (98–100%), and malignant peripheral nerve sheath tumors (29–50%), and has also been detected in myoepitheliomas, granular cell tumors, and mammary carcinomas, among others (19, 20). STS are much less common in humans than in dogs and, as such, are rarely considered as a differential for spindloid amelanotic melanomas. The use of SOX-10 IHC for the diagnosis of OMM in dogs had not yet been validated. In our study, SOX-10 IHC had 100% sensitivity but only 90% specificity, and, similarly, there was 100% sensitivity and only 85% specificity using relative *SOX10* RNA levels to discriminate between OMM and STS. The evaluation of SOX-10 expression by both IHC and quantitation of *SOX10* RNA expression in routine veterinary diagnostics is of limited use for diagnosing melanomas, similar to the evaluation of S100 or MITF expression (5). The lack of immunoreactivity for SOX-10 may be useful to exclude a melanocytic neoplasm, but labeling for SOX-10 should not be used as a single criterion for confirmation of a diagnosis of OMM. While in our study only 2/20 STS had immunoreactivity for SOX-10 and an additional tumor expressed *SOX10* RNA levels above the cutoff, five cases in the OSCT group had a strong immunoreactivity for SOX-10 and *SOX10* RNA levels above the cutoff. This difference in the percentage of cases in which SOX-10 expression was identified between STS and OSCT raises concern that our selection of STS may have been biased toward less differentiated tumors, leading to an underestimation of the number of SOX-10-positive STS. The reason for this speculation is that none of the five OSCTs with immunoreactivity for SOX-10 and *SOX10* RNA levels above the cutoff had expression levels of *TYR* RNA above the cutoff, which does not support a diagnosis of OMM. Furthermore, the RNA levels of *CALD1* and *CD34* were above the cutoffs for two of these five OSCT. There was 100% specificity for a diagnosis of STS using an evaluation of *CALD1* and *CD34* RNA expression levels; following this paradigm, having the expression of *CALD1* and *CD34* RNA above the cutoff levels for detection excluded a diagnosis of OMM for these two cases. Lastly, one of the remaining three OSCTs with immunoreactivity for SOX-10 and *SOX10* RNA levels above the cutoff had RNA expression levels for *DES* RNA above the cutoff, and all three cases were immunohistochemically positive for laminin and negative for S100, which are features supportive of a diagnosis of STS.

To summarize, of the 20 OSCT cases, there were only five that could have been potentially diagnosed as OMMs based on the SOX-10 IHC and/or *SOX10* RNA data. Two of these were instead confirmed as STS based on *CALD1* and *CD34* RNA expression levels. For the remaining three cases, the lack of S100 labeling, expression of *DES* RNA above the cutoff in one case, and varying degrees of laminin labeling make a diagnosis of STS highly likely; therefore, these cases were diagnosed as suspect STS. One more case was diagnosed as a suspect STS, as it lacked an expression not only of *SOX10* and *TYR* RNA but also of *CALD1, CD34*, and *DES* RNA. In addition, it was negative for S100 and expressed low levels of laminin and low levels of SOX-10. An alternative classification of this case as an undifferentiated malignant neoplasm could be argued, considering the low numbers of IHC-positive cells.

Laminin is a key component of basement membranes and has been used as a marker for malignant peripheral nerve sheath tumors in dogs (9). Interestingly, in a minipig model of melanomas, neoplastic melanocytes were surrounded by the granular expression of laminin as evaluated by immunofluorescence (21). In humans, immunohistochemical labeling for laminin surrounding neoplastic melanocytes has been identified within the dermis (22). Neither RNA nor the protein expression of laminin has previously been investigated in canine OMMs. In our study, there was immunoreactivity for laminin in the pericellular matrix surrounding STS cells in 90% (18/20) of cases and surrounding neoplastic melanocytes in 30% (6/20) of cases. As such, the use of laminin IHC lacks the specificity needed to be a useful marker to discriminate between OMMs and STS. Additionally, the correlation between IHC and RNA levels for laminin was very poor, most likely due to sample heterogeneity within the analyzed portions of the mass. In general, sample heterogeneity may have a negative impact on both IHC results and RNA expression levels regardless of the target, especially when evaluating small punch biopsies. However, based on the consistency of RNA expression and IHC data in our study, the analysis of regular biopsy samples minimized the impact of sample heterogeneity for most evaluated targets, e.g., SOX-10.

Based on the combination of IHC and RNA expression results, OSCTs represent a highly heterogeneous group of neoplasms, with most representing soft tissue sarcomas. As determined by immunoreactivity for desmin and S100, two and five OSCT cases had myopericyte and nerve sheath cell differentiation, respectively. Despite the limitations of using laminin IHC as a diagnostic criterion as discussed above, six OSCT cases had a widespread laminin immunoreactivity (score 2–3), and half of these cases were also positive for S100 by IHC, supporting nerve sheath cell differentiation. All five S100 IHC-positive OSCTs also had RNA expression levels for *CD34* above the cutoff, supporting a differentiation toward a perivascular wall tumor phenotype. Interestingly, while one of the OSCTs that was immunoreactive for desmin also had an RNA level of *CALD1* above the cutoff, supporting myopericyte differentiation, the other desmin IHC-positive case had low *CALD1* RNA levels. Of the 12 OSCTs with *CD34* RNA expression levels above the cutoff, four also had *CALD1* RNA levels above the cutoff, and three had *DES* RNA levels above the cutoff. Most surprisingly, two OSCTs had RNA levels above the cutoffs for both *CALD1* and *DES*, and one of these two cases also had *CD34* RNA levels above the cutoff. It is unclear whether these results simply reflect the heterogeneity of the analyzed samples (e.g., high vascularity) or if such diverse expression patterns are a reflection of the reactivation of genes that encode different lines of mesenchymal differentiation by malignant mesenchymal cells (9).

Tyrosinase is a copper-containing membrane glycoprotein that represents a key enzyme in the initiation of melanogenesis (23). The expression of *TYR* RNA has been detected in human melanoma cell lines (10). As expected, *TYR* RNA expression was highly specific for the detection of OMM in this study, and none of the STS or OSCT expressed *TYR* RNA above the cutoff. While the high specificity of *TYR* RNA expression for the detection of OMM could be exploited as a future diagnostic test, a sensitivity of 65% was an unexpectedly low result. In a study in human melanomas, only 74% of metastatic melanomas expressed *TYR* RNA (10). The lack of pigmentation in amelanotic or metastatic melanomas has been suggested as the cause for the inability to detect *TYR* RNA (24). All OMMs in this study were poorly pigmented, and RNA was extracted from large tumor areas rather than from sites where neoplastic cells were pigmented. This explanation is further supported by the negative *TYR* RNA results for the two OMMs that were only MDX-positive within intraepithelial nests, as the epithelium was excluded from RNA extraction in this study. Increased copy number gains in 8q24 at *MYC* have been detected in 90% of cutaneous melanomas in humans (24). The oncogene c-myc has been shown to have upstream regulatory effects on melanogenesis through suppression of the microphthalmia-associated transcription factor (MITF), which regulates the expression of tyrosinase (25). The downregulation of *TYR* has been observed in amelanotic melanomas with gains in 8q24 as a result of this mechanism (24). A similar genetic alteration may have caused the loss of expression of *TYR* RNA in our study. As the immunogenic tyrosinase has been found to be overexpressed in malignant melanocytes, as compared to normal cutaneous melanocytes (26), vaccines against tyrosinase that elicit a cytotoxic T cell immune response that targets melanocytes expressing tyrosinase have been used successfully in humans (27). Similarly, an important therapeutic option for OMMs is the xenogeneic human tyrosinase DNA-based vaccine, ONCEPT® (28). Interestingly, in our study, only 13 OMMs had extremely high relative levels of *TYR* RNA (to the order of 10^5^), while seven cases had very low to imperceptible levels of relative RNA expression. An association between *TYR* RNA expression levels in canine OMM and responsiveness to the ONCEPT® vaccine has not been investigated. In a follow-up study, we intend to investigate a potential correlation between *TYR* RNA expression levels by OMMs and their responsiveness to the ONCEPT® canine melanoma vaccine.

## Conclusion

In conclusion, this study determined that relative RNA expression levels of *TYR, CD34*, and *CALD1* discriminate between canine oral melanomas and soft tissue sarcomas. Moreover, RT-qPCR for the RNA expression of *TYR, CD34*, and *CALD* may be a useful diagnostic tool following a negative MDX result in suspected spindloid amelanotic OMM cases that lack an overlying epithelium.

## Data Availability Statement

The original contributions presented in the study are included in the article/Supplementary Material, further inquiries can be directed to the corresponding author/s.

## Author Contributions

MT generated, analyzed, interpreted data, and drafted the manuscript. TT created the study concept and design, generated, analyzed, interpreted data, and revised the manuscript. RS and MK created the study concept and design, interpreted data, and revised the manuscript. EN created the study concept and revised the manuscript. All authors contributed to the article and approved the submitted version.

## Conflict of Interest

The authors declare that the research was conducted in the absence of any commercial or financial relationships that could be construed as a potential conflict of interest.

## Publisher's Note

All claims expressed in this article are solely those of the authors and do not necessarily represent those of their affiliated organizations, or those of the publisher, the editors and the reviewers. Any product that may be evaluated in this article, or claim that may be made by its manufacturer, is not guaranteed or endorsed by the publisher.
