# Supplementary material for: Quantitative Expression of TYR, CD34, and CALD1 Discriminates Between Canine Oral Malignant Melanomas and Soft Tissue Sarcomas
Source: Front Vet Sci. 2021 Aug 6;8:701457. doi: 10.3389/fvets.2021.701457 (PMC8377394; doi:10.3389/fvets.2021.701457)
Supplement: Supplementary file 1 [file Data_Sheet_1.docx]

Supplemental Material

**Supplemental Table 1. List of gene names, gene locations, and primers used for amplification**

| **Gene** | **Source** | **Forward primer** | **Reverse Primer** | **Size**  **(base pairs)** |
| --- | --- | --- | --- | --- |
| B2M | ENSCAFG00000013633 | CCTTGCTCCTCATCCTCCTC | ACCCTGACACGTAGCAGTTCA | 129 |
| TYR | NM_001002941 | TCCAGAGATCTGGGCTATGAC | AGTGACGACACAGCCTACCAC | 144 |
| SOX10 | ENSCAFT00000002231.3 | AGTACCCGCACCTGCACAA | GCTCAGCCTCCTCAATGAA | 102 |
| CALD1 | ENSCAFG00000003169.5 | GCTAACTAAAACCCCAGATGGA | GATTGCTTTTCCCAGAGGTTC | 102 |
| CD34 | ENSCAFG00000011722.4 | TGAACCAAGGTATCTGCCTAGA | TCCTTCTCACACAGGACTTGG | 100 |
| DES | NM_001012394 | CAGCCAACAAGAACAACGAC | CTGCCTCATCAGGGAATCAT | 128 |
| LAMA1 | ENSCAFG00000018597.3 | GTGCCAAAGTAGATGCCATTG | AAGTGTGTGCCATTTTCCATC | 140 |

**Supplemental Table 2. Antibody sources, concentrations, retrieval, and controls**

| **Antibody** | **Source** | **Company** | **Concentration** | **Retrieval** | **Control** |
| --- | --- | --- | --- | --- | --- |
| Desmin | Mouse monoclonal | DAKO | 1:400 | HI, 20 min | Skeletal muscle |
| Laminin-1 | Rabbit polyclonal | DAKO | 1:750 | PK, 10 min | Skin |
| Melan-A | Mouse monoclonal | Novocastra | 1:100 | PT Low, 30 min | Melanoma |
| PNL2 | Mouse monoclonal | Santa Cruz | 1:500 | PT Low, 30 min | Melanoma |
| TRP-1 | Rabbit polyclonal | Aviva | 1:5000 | PT Low, 30 min | Melanoma |
| TRP-2 | Rabbit polyclonal | ABCAM | 1:500 | PT Low, 30 min | Melanoma |
| S100 | Rabbit polyclonal | Dako | 1:800 | None | Brain |
| SOX10 | Rabbit monoclonal | Cell Marque | 1:100 | PT Low, 30 min | Melanoma |

HI: heat inactivation; PK: proteinase K; PT: pre-treatment.
